# Beneficial effects of silibinin on serum lipids, bile acids, and gut microbiota in methionine-choline-deficient diet-induced mice

**DOI:** 10.3389/fnut.2023.1257158

**Published:** 2023-10-06

**Authors:** Wei Wang, Ting Zhai, Ping Luo, Xiaolei Miao, Junjun Wang, Yong Chen

**Affiliations:** ^1^Hubei Province Key Laboratory of Biotechnology of Chinese Traditional Medicine, National and Local Joint Engineering Research Center of High-throughput Drug Screening Technology, State Key Laboratory of Biocatalysis and Enzyme Engineering, Hubei University, Wuhan, China; ^2^School of Pharmacy, Hubei University of Science and Technology, Xianning, China

**Keywords:** silibinin, nonalcoholic steatohepatitis, methionine-choline-deficient diet, LC-MS, 16S rDNA sequencing

## Abstract

**Background and purpose:**

Silibinin (SIL) is a flavonoid lignin isolated from the fruit and seeds of silybum marianum that exhibits good therapeutic potential for NASH. However, the effects of SIL on serum lipids, bile acids (BAs), and gut microbiota (GM) in NASH mice remain unknown. The present work aimed to explore the beneficial effects of SIL supplementation on serum lipids, bile acids, and gut microbiota in MCD mice.

**Experimental approach:**

After male C57BL/6 mice were fed with a methionine-choline deficient (MCD) diet and simultaneously gavaged with SIL (20 mg/kg. d) for 8 weeks, the pathological changes of liver tissue were observed by oil red O, haematoxylin-eosin, and Masson tricolor staining; the levels of serum AST and ALT, and liver TG and MDA were detected by assay kits; metabonomics and 16S rDNA sequencing were used to analyze the composition of serum lipids and BAs and the abundance of GM; and the mRNA expression levels of hepatic genes related to BAs homeostasis were detected by RT-qPCR.

**Results:**

The results indicated that SIL treatment decreased the levels of 26 lipids (including four arachidonic acids, seven FFAs, 12 acyl carnitines, and three GPs) and two BAs (23-DCA, GLCA), while *Dubosiella* increased the levels of 10 lipids (including TxB3, PG16:0_18:1, Cer t18:0/24:0 and 7 TGs), five BAs (β-MCA, α-MCA, UDCA, 3-oxo-DCA and HCA), and two GMs (*Verrucomicrobiota* and *Akkermansiaceae*) of MCD mice, but had no significant effect on the mRNA expression of CYP7A1, CYP27A1, Bsep, Mrp2, Ntcp, or Oatp1b2. Therefore, influencing GM composition and then regulating the levels of serum lipids and BAs through enterohepatic axis should be an important mechanism of SIL-induced alleviative effect on MCD mice. More importantly, we found that SIL had a good coordination in regulating the abundance of GM and the contents of serum lipids and BAs in MCD mice, that is, when the abundance of probiotics was up-regulated, the content of beneficial unsaturated fatty acids in serum was up-regulated, while the serum levels of harmful lipids and BAs were down-regulated.

**Conclusion:**

The alleviating effect of SIL on NASH may be closely related to the correction of intestinal bacteria disorder, serum bile acid, and lipid metabolic disturbance in mice.

## Introduction

NAFLD is characterized by a large number of hepatocyte steatosis in the absence of alcohol or low alcohol consumption, which gradually progresses from simple steatosis to non-alcoholic steatohepatitis (NASH), and then to liver fibrosis, cirrhosis, and its ensuing complications. The pathogenesis of NAFLD is complex, but the accumulation of triglycerides (TG) in the liver is the first step in the development of NAFLD (1). On the one hand, some highly expressed fatty acid transporters, fatty acid binding proteins, and caveolins in the liver promote the uptake of free fatty acids (FFAs) by liver cells and accelerate lipid accumulation in the liver (2). On the other hand, hepatocytes mainly rely on the β oxidation of fatty acid for energy; excessive uptake of FFAs impairs mitochondrial oxidative phosphorylation, and incomplete FFAs oxidation can also promote the synthesis and accumulation of toxic lipid intermediates, such as ceramides and diacylglycerol (3). In addition, excessive FFAs can activate the IKK-β/NF-κB pathway by blocking the signaling pathway for insulin receptor substrate-1 and induce apoptosis of pancreatic islet B cells to trigger insulin resistance (IR), which in turn promotes fat synthesis and storage in the liver (4).

Farnesoid X receptor (FXR), which is expressed in the liver and intestine, is the main target gene that regulates the expression of genes related to the synthesis and transport of bile acids (BAs) and is a crucial element for maintaining the homeostasis and enterohepatic circulation of Bas (5). BAs are the end-product of cholesterol metabolism. The enterohepatic circulation of BAs plays an important role in the emulsification and intestinal absorption of lipids and other nutrients. Recent studies have found that BAs, as the signaling molecules, can regulate FXR activity not only by inhibiting the expression of small heterodimeric protein (SHP)-mediated sterol regulatory element binding protein 1c (SREBP-1c) and inducing FXR-dependent peroxisome proliferation activation receptor α (PPARα) expression to reduce liver fatty acid synthesis and promote fatty acid β-oxidation, but also by promoting the secretion of FGF15 (mice)/FGF19 (human) by ileal cells and then combining with FGFR4 receptor expressed by hepatocytes after circulation through the portal vein to inhibit the expression of CYP7A1 and regulate the synthesis of bile acids (6). Disorders of the gut microbiota (GM) is strongly associated with NAFLD, for example, *Bacteroides* is associated with liver inflammation and *Ruminococcus* is associated with liver fibrosis (7). Intestinal dysbacteriosis leads to NAFLD through several possible mechanisms, including increasing the production and absorption of intestinal short-chain fatty acids, affecting the transformation of dietary choline, altering intestinal permeability, promoting bacterial endotoxin release (8), and changing the pool size and composition of BAs (9).

Silibinin (SIL) is a flavonoid lignin isolated from the fruits and seeds of *silybum marianum*, which is from the asteraceae family, and exhibits liver protection, antioxidant, antitumor, and liver cell membrane stability maintenance properties (10, 11). Studies found that SIL reduced lipid peroxidation, plasma insulin, and TNF-α levels of high-fat diet (HFD)-induced NASH rats (12), alleviated liver lipid metabolism disorder and inflammation of methionine-choline deficient (MCD) diet-induced mice (13), reduced the consumption of hepatic GSH, and the production of mitochondrial hydrogen peroxide and hepatic human neutrophil elastase (HNE) in MCD rats (14), and alleviated IR and steatosis of palmitic acid-induced BRL3A and HepG2 cells (15). Our previous results showed that SIL alleviated NASH in MCD mice by activating the CFLAR-JNK pathway to up-regulate the expression of hepatic lipid oxidation and lipid transport-related genes, and to activate NRF2 to regulate the expression of its downstream antioxidant factors (16).

Due to the important roles of lipids, BAs, and GM in the occurrence, development, and regression of NASH, the effects of SIL on the composition of serum lipids and bile acids, and ileal microbiota in MCD mice, were studied in this work by using metabonomics and 16SrDNA sequencing techniques.

## Materials and methods

### Drugs and reagents

Silibinin and obeticholic acid were purchased from Shanghai Yuanye Bio-Technology Co., Ltd. (purity ≥ 98%). Methionine- and choline-deficient feed (MCD) and whole nutrient control feed (MCS) were purchased from Trophic Animal Feed High-Tech Co., Ltd, China (Jiangsu, China). Methanol, acetonitrile, isopropanol, methyl tert-butyl ether (chromatographic pure, Merck), ammonium formate, methylene chloride (MS pure, Fisher), formic acid, acetic acid, and ammonium acetate (chromatographic pure, Sigma–Aldrich); Standard 12:0 Lyso PC, Cer (d18:1/4:0), PC (13:0/13:0), DG (12:0/12:0), TG (17:0/17:0/17:0) were purchased from Avanti/zzstandard; 50 bile acid standards were purchased from CNW/IsoReag; the buffer Phusion^®^ High-Fidelity PCR Master Mix with GC Buffer was purchased from New England Biolabs, and RNaseA (RNaseA) was purchased from Promega. Alanine aminotransferase (ALT), aspartate aminotransferase (AST), triglyceride (TG), and malondialdehyde (MDA) test kits were purchased from Nanjing Jiancheng Institute of Bioengineering (Nanjing, China). TRIzol was purchased from Invitrogen Corporation (Carlsbad, CA, USA). The reverse transcription kit ReverTra Ace qPCR RT Master Mix with gDNA Remover kit was purchased from Toyobo Toyobo Co., Ltd. (Life Science Department OSAKA JAPAN). The SYBR Green I fluorescent quantitative PCR kit was purchased from Bio-Rad (Hercules, CA, USA).

### Animal experiments

SPF-rated male C57BL/6 mice, weighing 19–23 g and aged 6–8 weeks, were purchased from the Hubei Provincial Center for Disease Control and Prevention. Mice were housed and maintained in a clean animal room under the conditions of temperature 22 ± 2°C, relative humidity 55–65%, and 12 h light and dark each day. During the feeding process, mice were fed and drank freely. After 7 days of adaptive feeding, mice were randomly divided into normal control group, model group, SIL (20 mg kg-1 d-1) group, and obeticholic acid (OCA, 6.5 mg kg-1 d-1) positive control group, with seven mice in each group. The gavage dose of SIL was set as the middle dose of our previous research work (16), and the gavage dose of obeticholic acid was 6.5 mg/kg.d which was converted according to the recommended oral dose for adults (5 mg once daily). Mice in the normal control group were fed with an MCS diet; mice in other groups were fed with an MCD diet. Additionally, the SIL group and OCA group were, respectively gavaged SIL and OCA once a day, while normal control and model groups were gavaged with the equal volume of 0.5% CMC-Na solution once a day, which was sustained for 8 weeks. The weight of mice was measured once a week. After the last dose, mice were fasted for 12 h, and then blood was extracted from the ocular venous plexus to prepare serum (4°C, 3,500 rpm min-1 centrifugation for 15 min) and stored in a −80°C freezer. Liver tissue and ileal contents were collected after mice were sacrificed. All experiments were approved by the Experimental Animal Ethics Committee of Hubei University and followed the principles of humanity.

### Liver tissue histopathology and serum and liver biochemical analysis

Approximately 1 cm^3^ of fresh liver was taken from each mouse, fixed with 4% paraformaldehyde for 24 h, dehydrated and embedded with paraffin, and then cut into 5 μm thin slices for hematoxylin-eosin (HE), Masson tricolor, and oil red O staining to evaluate the pathological condition of liver histopathology.

The kits were used to determine ALT and AST activity in serum, as well as TG and MDA content in liver tissue homogenate (liver/normal saline = 1/9, w/w), as per the manufacturer's instructions.

### Serum lipidomics analysis

#### Sample treatment

Serum (50 μL) from each mouse was mixed with 1 mL of lipid extract solution (methyl tert-butyl ether/methanol, 3/1, V/V) containing internal standard, and vortexed for 15 min. Then, 200 μL of pure water was added and the mixture was vortexed for 1 min and centrifuged (12,000 r min ^−1^) at 4°C for 10 min. A total of 200 μL of supernatant was collected and concentrated to dry, redissolved using 200 μL of acetonitrile/isopropanol (10/90, V/V), and centrifuged (12,000 r min −1) for 3 min. The supernatant was collected for LC-MS analysis. Quality control sample (QC) was prepared by mixing equal amounts of extracts from each sample. During the analysis, a QC was inserted for every 10 samples tested to monitor the repeatability of the analysis process.

#### Untargeted serum lipidomics analysis

Exion LC AD UPLC-QTRAP (SCIEX, USA) was used to detect the composition and relative content of serum lipids. The column was a Thermo Accucore™ C30 (2.1 x 100 mm, 2.6 μm), and the mobile phases consisted of A (acetonitrile/water, 60/40, V/V, containing 0.1% formic acid and 10 mmol L-1 ammonium) and B (acetonitrile/isopropanol, 10/90, V/V, containing 0.1% formic acid and 10 mmol L-1 ammonium formate). The gradient elution procedure was: 0–2 min, 30% B; 2–4 min, 60% B; 4–9 min, 85% B; 9–14 min, 90% B; 14–15.5 min, 95% B; 17.3–17.5 min, 20% B; and 17.5–20 min, 20% B. The flow rate was 0.35 mL min^−1^. The column temperature was 45°C. The injection volume was 2 μL. Positive and negative ion scanning (m/z 100–1,500) was performed using an electrospray ionization source under the conditions of ion source temperature of 500°C, mass spectrometry voltage of 5,500 V (positive ion)/−4,500 V (negative ion), ion source gas 1 (GS1) of 45 psi, gas 2 (GS2) of 55 psi, and curtain gas (CUR) of 35 psi.

After the lipidomics' detection data were extracted and pre-processed by Analyst 1.6.3 software, multivariate statistical analysis was performed using R software (www.r-project.org/), including unsupervised principal component analysis (PCA) and supervised orthogonal partial least squares discriminant analysis (OPLS-DA), to determine the difference in lipid sample distribution between groups. Lipid species with significantly different levels were screened according to the OPLS-DA variable importance (VIP ≥ 1) and the fold change (≥2 and ≤ 0.5) of univariate analysis, and the clustering heatmap was used to visualize them. Additionally, the differential lipid components were introduced into the KEGG (Kyoto Encyclopedia of Genes and Genomes) database for metabolic pathway enrichment analysis.

### Serum bile acid analysis

Exion LC AD UPLC-QTRAP 6,500+ (SCIEX, USA) was used to determine the content of 50 serum BAs. The column was Waters ACQUITY UPLC HSS T3 C18 (1.8 μm, 100 mm × 2.1 mm) with mobile phases A (ultrapure water containing 0.01% acetic acid and 5 mmol L-1 ammonium acetate) and B (acetonitrile containing 0.01% acetic acid). The gradient elution procedure was as follows: 0 min, 5% B; 0–0.5 min, 40% B; 0.5–4.5 min, 50% B; 4.5–7.5 min, 75% B; 7.5–10 min, 95% B; and 10–12 min, 5% B. Flow rate was 0.35 mL min ^−1^. The column temperature was 40°C and the injection volume was 3 μL. The samples underwent electrospray ionization (ESI) in negative ion mode and were detected in preset multireaction monitoring (MRM) mode under the conditions of ion source temperature of 550°C, mass spectrometry voltage of −4,500 V, source gas of 45 psi, gas 2 of 55 psi, and curtain gas (CUR) of 35 psi.

### 16S rDNA sequencing analysis of gut microbial diversity

The ileal contents of each group of mice were collected, the microbial genomic DNA was extracted by the CTAB method, the purity and concentration of DNA were detected by 0.5% agarose gel electrophoresis, and an appropriate amount of DNA samples were collected in centrifuge tubes and diluted with sterile water to 1 ng μl-1 as a template. Depending on the selected sequencing region, PCR was performed using the specific primers with Barcode (515F and 806R), High-Fidelity PCR Master Mix with GC Buffer, and highly efficient high-fidelity enzymes to amplify the 16S V4 region. After the quality of the PCR product was detected by 2% agarose gel electrophoresis, the qualified PCR product was recovered with the Gel Extraction Kit (Qiagen), and then was collected by magnetic bead purification and microplate quantification. The library was constructed with the TruSeq^®^ DNA PCR-Free Sample Preparation Kit. Qubit and Q-PCR were used to detect library eligibility, and NovaSeq6000 sequenced the qualified library. According to the overlap relationship splicing sequencing, pair-end reads were obtained, the barcode sequence was removed, and the effective sequence was generated after performing statistical analysis with the number and length distribution of the sequence. According to the similarity of the sequences, sequences with a similarity of more than 97% were classified into multiple operational taxonomic units (OTUs) and subsequently analyzed.

### RT–qPCR

The total RNA of mouse liver (50–100 mg) was extracted by a TRIzol kit (Invitrogen, Carlsbad, CA, USA), the integrity of RNA was detected by 0.1% agarose gel electrophoresis, and the purity and concentration of RNA were measured spectrophotometrically. RNA was transcribed to cDNA using the ReverTra Ace^®^ qPCR RT kit (Toyobo, Japan), and real-time Quantitative PCR (RT-qPCR) using the SYBR Green fluorescent quantitative PCR kit and CFX Connect™ Real-Time System (BIO-RAD, USA) was performed under the conditions of predenaturation at 95°C for 5 min, denaturation at 95°C for 30 s, annealing at 56.3°C for 30 s, and extension at 72°C for 30 s. β-actin was used as the internal reference gene. The relative expression of mRNA of each gene was calculated by the 2–ΔΔCt method. Table 1 shows the primer sequences of RT-qPCR.

**Table 1:** Primer sequences used for RT-qPCR.

**Name**	**Accession number**		**Sequences**
*β-actin*	NM_007393	Forward Reverse	5′-AGAGGGAAATCGTGCGTGAC-3′ 5′-CAATAGTGATGACCTGGCCGT-3′
*CYP7A1*	NM_007824	Forward Reverse	5′-GGGAATGCCATTTACTTGGA-3′ 5′-GTCCGGATATTCAAGGATGC-3′
*CYP27A1*	NM_024264	Forward Reverse	5′-CTATGTGCTGCACTTGCCC-3′ 5′-GGGCACTAGCCAGATTCACA-3′
*Bsep*	NM_021022	Forward Reverse	5′-CCAGAACATGACAAACGGAA-3′ 5′-AAGGACAGCCACACCAACTC-3′
*Mrp2*	NM_013806	Forward Reverse	5′-TCCAGGACCAAGAGATTTGC-3′ 5′-TCTGTGAGTGCAAGAGACAG GT-3′
*Ntcp*	NM_011387	Forward Reverse	5′-AGGGGGACATGAACCTCAG-3′ 5′-TCCGTCGTAGATTCCTTTGC-3′
*Oatp1b2*	NM_020495	Forward Reverse	5′-ACCAAACTCAGCATCCAAGC-3′ 5′-TAGCTGAATGAGAGGGCTGC-3′

### Data analysis

The results of this experiment were analyzed using SPSS 25.0 statistical software and are expressed as the mean ± SD. The comparison between the two groups was performed using a *t*-test for two independent samples, the comparison between the groups was performed using one-way ANOVA, and *p* < 0.05 was considered statistically significant.

## Results

### Effects of SIL on body weight and liver index in mice

**Figure 1:**
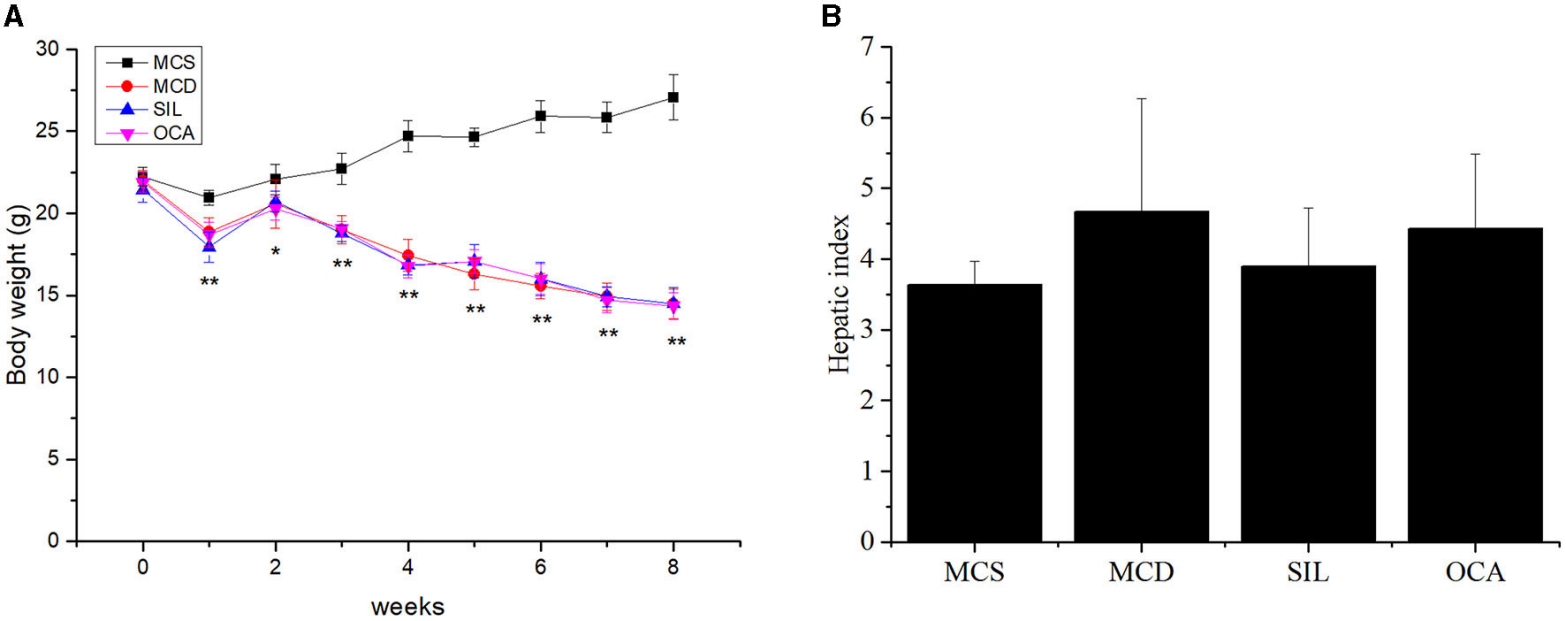
Effects of SIL on body weight and hepatic index of MCD mice. **(A)** Body weight. **(B)** Liver index (liver weight/body weight × 100). *n* = 7. *p < 0.05, **p < 0.01 vs. MCS group.

The weight of MCD mice gradually decreased over time, and there was no significant difference in the average body weight of mice in MCD, SIL, or OCA groups (Figure 1A). At the eighth week, there was no significant difference in the liver index of mice in MCS, MCD, SIL or OCA groups (Figure 1B).

### Effects of SIL on the pathological changes of liver tissues in MCD mice

**Figure 2:**
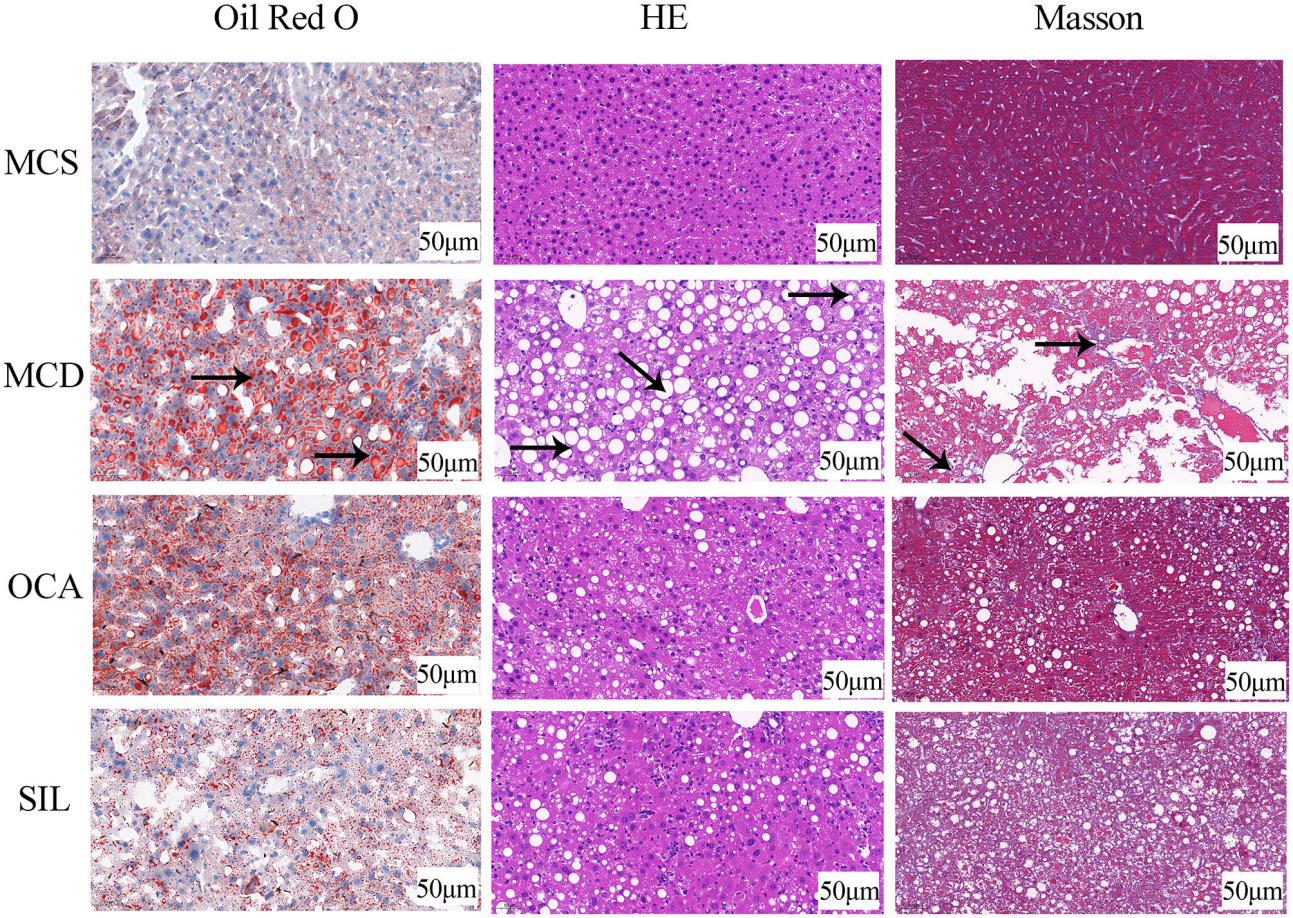
Effects of SIL on the pathological changes of liver tissues in MCD mice. Arrow ( → ) indicates the corresponding pathological changes.

Figure 2 showed the pathological changes of liver tissues in the tested mice by Oil red O staining, HE staining, and Masson staining. Compared with the MCS group, lipid droplets in the liver cells of MCD mice were significantly increased and the arrangement of liver cells was disordered with a balloon-like transformation and a large number of fat vacuoles, accompanied by inflammatory cell infiltration and local fibrosis. After OCA or SIL treatment, the above conditions were improved to some extent, and the liver morphology tended to be normal. In addition, SIL had no obvious effect on liver fibrosis, while OCA had a certain effect on liver fibrosis.

### Effect of SIL on serum and liver biochemical indices in mice

**Figure 3:**
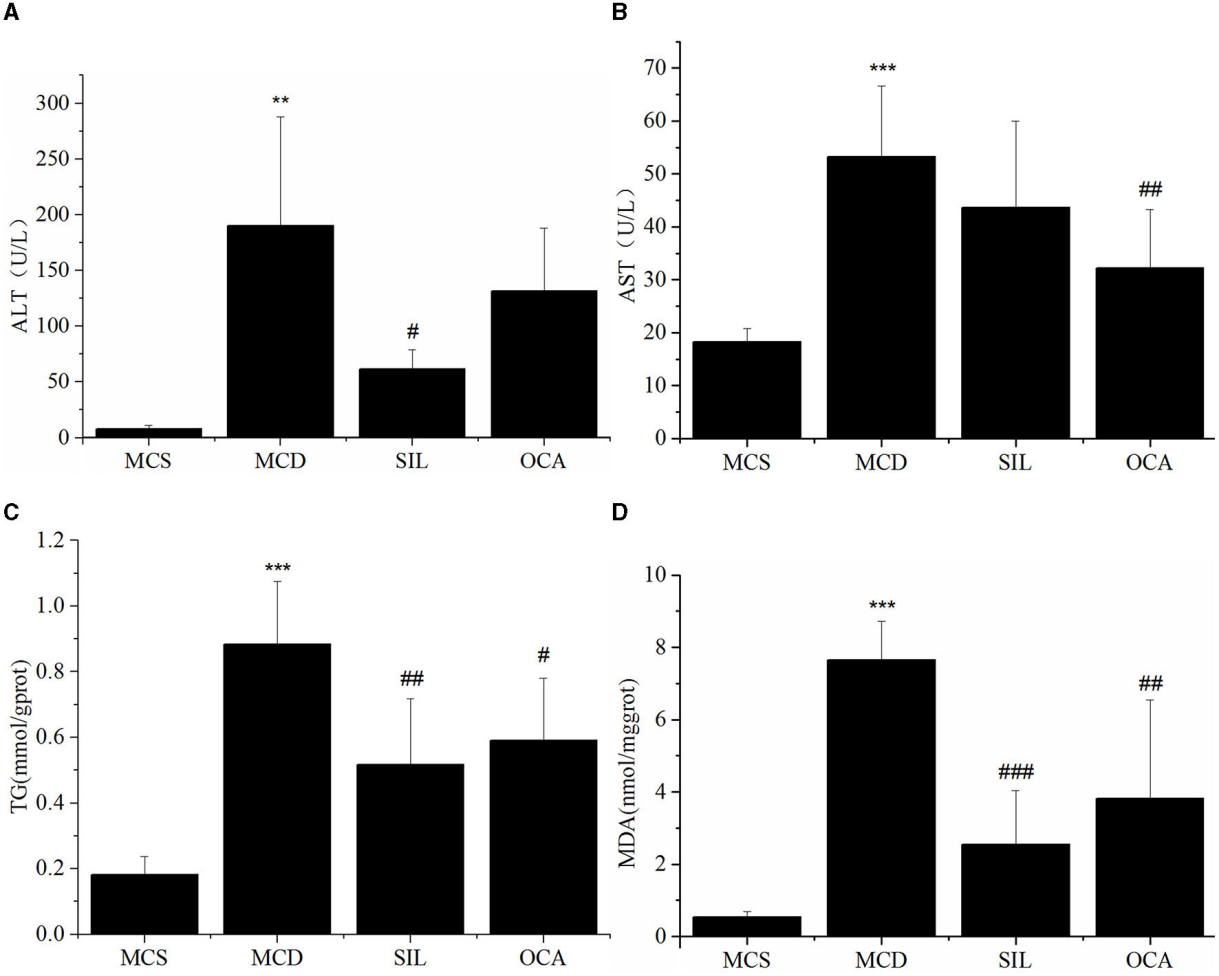
Effect of SIL on the hepatic injury-related biomarkers of the tested mice. **(A)** Serum ALT activity. **(B)** Serum AST activity. **(C)** Liver TG level. **(D)** Liver MDA level. *n* = 7. *p < 0.05, **p < 0.01 vs. MCS group, #*p* < 0.05, ##*p* < 0.01, ###*p* < 0.01 vs. MCD group. ***p < 0.001 indicates statistically significant between MCS and MCD group.

The levels of serum ALT and AST and liver TG and MDA in each group are shown in Figure 3. The results showed that the levels of ALT, AST, TG, and MDA in the MCD group were significantly higher than those in the MCS group, and the levels of ALT, TG and MDA, in the SIL group were significantly lower than those in the MCD group. Although the AST level showed a downwards trend, there was no significant difference compared with the MCD group. In addition, the levels of AST, TG, and MDA in the OCA group were significantly lower than those in the MCD group.

### Effect of SIL on the composition and relative content of serum lipids in MCD mice

**Figure 4:**
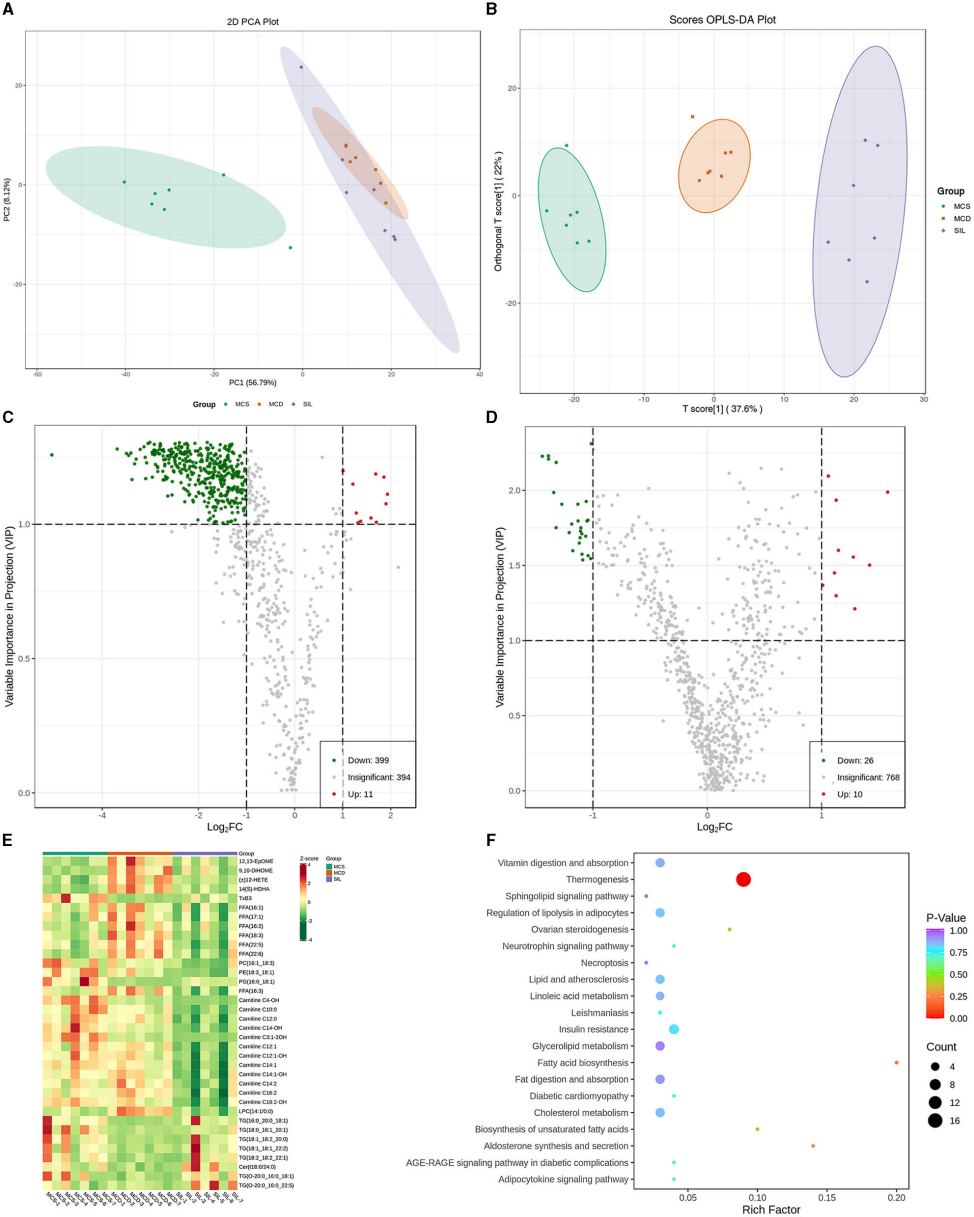
Differential lipid species among MCS, MCD, and SIL groups (*n* = 7). **(A)** PCA score plot. **(B)** OPLS-DA score between MCS, MCD, and SIL groups. **(C)** Volcano plots of lipid species between MCD group and MCS group. **(D)** Volcano plots of lipid species between MCD group and SIL group. **(E)** Heat map for hierarchical cluster analysis of differential lipids. **(F)** KEGG pathway enrichment analysis of 36 differential lipids between MCD group and SIL group.

PCA showed the trend of lipid separation in each group, with each point representing a sample (Figure 4A). The results indicated that the sample distribution of the MCS and MCD group exhibited a significant separation trend, and the sample distribution of the SIL group was closer to that of the MCS group than the MCD group. OPLS-DA can also determine the separation of samples between groups and determine whether the method is valid with two main parameters, namely R2Y (representing the explanatory rate of the model) and Q2 (representing the prediction rate of the model). Figure 4B showed the OPLS-DA scores of serum lipid distribution in MCS, MCD, and SIL groups. The values of R2Y and Q2 (R2Y = 0.986, Q2 = 0.794) were >0.4, indicating that the sample distribution among the three groups could be effectively separated by OPLS-DA.

The volcanic map of OPLS-DA more intuitively showed the differential lipid types among the groups (Figures 4C, D). The green dot in the map represented the down-regulated lipids, and the red dot represented the up-regulated lipids. According to the variable importance in projection (VIP) value and difference multiple values of lipid comparison in each group, there were 410 differential lipid metabolites between the MCS group and MCD group, 399 of which were down-regulated and 11 were up-regulated, including 23 fatty acyls (FAs), 281 glycerophospholipids (GPs), 39 sphingolipids (SPs), 14 sterol lipids (STs), and 53 glycerides (GLs). Compared with MCD group, the SIL group contained 36 differential lipid metabolites, among them, four arachidonic acids [12,13-EpOME, 9,10-DiHOME, (±) 12-HETE, 14 (S) – HDHA], seven FFAs (16:1, 17:1, 16:2, 18:3, 22:5, 22:6, 16:3), 12 acyl carnitines (AC-C4-OH, - C10:0, - C14:0, - C14-OH, - C3:1-2OH, - C12:1, - C12: 1-OH, - C12: 1-OH, - C12: 1-OH, - C12: 1-OH, - C14:1, - C14:1-OH, - C16:2, - C18:2-OH), and three GPs (PC16:1_18:3_18:1, PE18:3_18:1, LPC14:1/0:0) that were down-regulated, and one arachidonic acid (TxB3), one GP (PG16:0_18:1), 7 TG (16:0_20_0_18:1, 18:0_18:1_20:1, 18:1_18:2_20:0, 18:1_18:1_22:2, 18:2_18:2_22:1, O-20:0_16:0_18:1, O-20:0_16:0_22:5), and one SP (Cer t18:0/24:0) that were up-regulated. The levels of 36 differential lipids mentioned above in the SIL group were closer to those in the MCS group. Figure 4E shows the heatmap for the cluster analysis of differential lipid levels between the MCD and SIL group.

The metabolic pathways related to the above 36 differential lipids were determined by enrichment analysis of KEGG pathways (Figure 4F). The results showed that there were 20 metabolic pathways between SIL and MCD groups, and only thermogenesis was related to the regulation of lipid metabolism disorder by SIL in MCD mice.

### Effect of SIL on serum bile acids in mice

The significant changes in serum BA levels between the three groups of mice are shown in Table 2. Compared with the MCS group, the levels of omega-muricholic acid (ω-MCA), 7-ketodeoxycholic acid (7-KDCA), β-MCA, deoxycholic acid (DCA), chenodeoxycholic acid (CDCA), α-MCA, 23-nor-deoxycholic acid (23-norDCA), 3- oxodeoxycholic acid (3-oxo-DCA), lithocholic acid (LCA), ursocholic acid (UCA), hyocholic acid (HCA), 3-oxocholic acid (3-oxo-CA), and glycolithocholic acid (GLCA) were significantly increased, and ursodeoxycholic acid (UDCA) content was significantly reduced in the MCD group. Compared with the MCD group, the levels of β-MCA, α-MCA, UDCA, 3-oxo-DCA, and HCA were significantly increased, while the levels of 23-norDCA and GLCA were significantly reduced in the SIL group.

**Table 2:** Effect of SIL on the levels of bile acids in serum of the tested mice.

	**MCS**	**MCD**	**SIL**
ω-MCA	1883.20 ± 994.81	5445.59 ± 1817.29^**^	5327.92 ± 4010.21
7-KDCA	870.27 ± 683.27	3828.77 ± 1181.29^**^	5772.32 ± 4126.03
β-MCA	818.50 ± 867.35	2846.72 ± 1490.44^*^	6160.43 ± 3032.19^#^
DCA	307.32 ± 163.26	669.23 ± 201.53^**^	445.02 ± 249.12
CDCA	238.98 ± 175.23	681.65 ± 261.41^**^	857.48 ± 425.82
α-MCA	108.37 ± 112.26	284.29 ± 135.01^*^	586.24 ± 290.72^#^
UDCA	53.38 ± 24.87	31.66 ± 27.82^*^	216.38 ± 96.61^##^
23-norDCA	27.14 ± 16.95	269.79 ± 41.87^**^	133.04 ± 62.24^##^
3-oxo-DCA	19.18 ± 13.37	54.78 ± 21.05^*^	50.59 ± 63.44
LCA	17.86 ± 4.11	27.39 ± 2.66^**^	26.55 ± 8.52
UCA	15.04 ± 5.66	111.91 ± 54.98^**^	130.84 ± 120.08
HCA	14.30 ± 10.71	71.18 ± 23.16^**^	112.09 ± 53.35^#^
3-oxo-CA	3.81 ± 3.73	8.82 ± 2.44^*^	16.89 ± 11.12^#^
GLCA	1.47 ± 0.14	1.86 ± 0.30^*^	1.41 ± 0.34^#^

All data are presented as the means ± SD. *n* = 7. ^*^p < 0.05, ^**^p < 0.01 vs. MCS group, #*p* < 0.05, ##*p* < 0.01 vs. MCD group.

**Figure 5:**
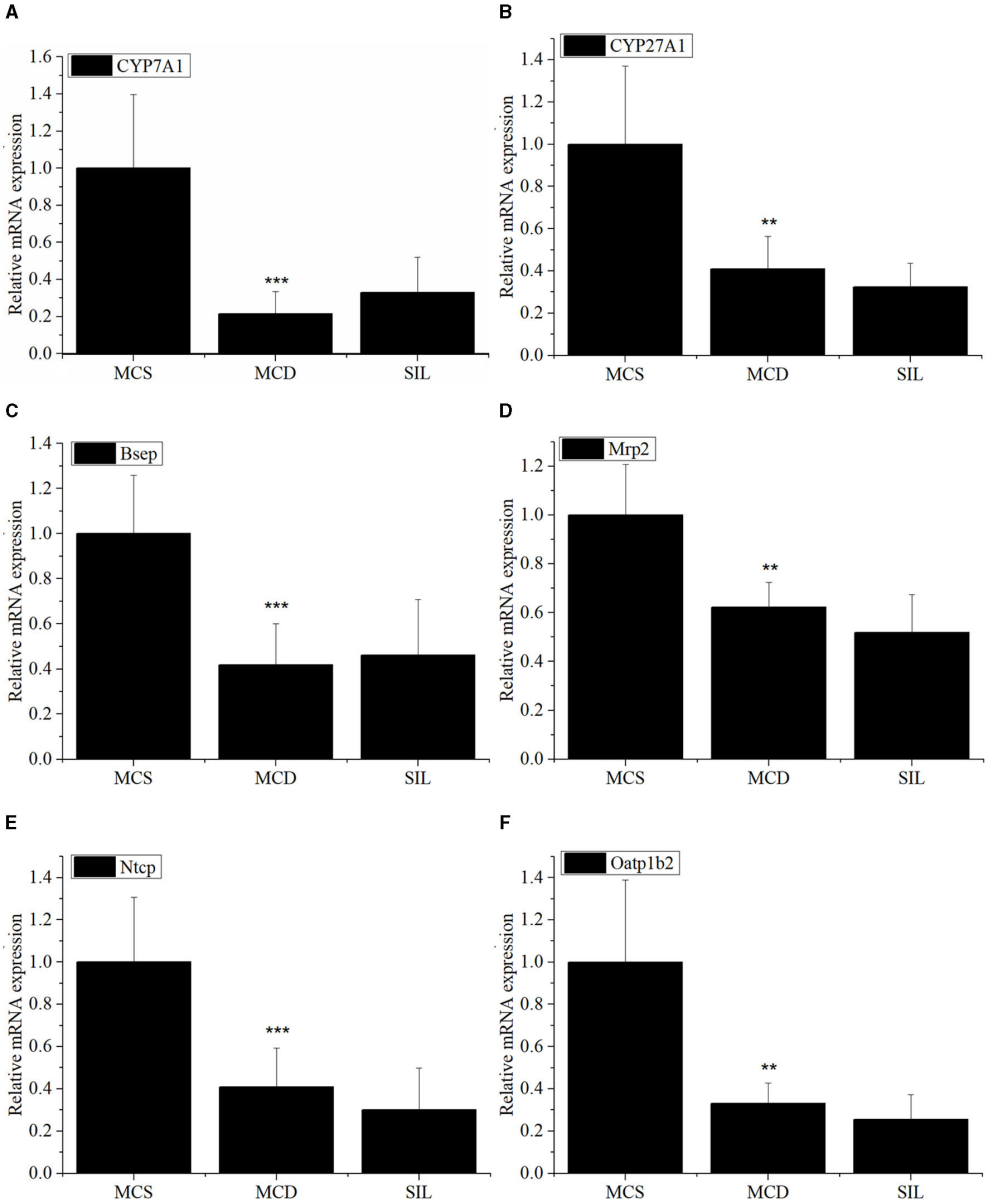
Effects of SIL on the mRNA expression of BA metabolism-related genes *CYP7A1* **(A)**, *CYP27A1* **(B)**, *Bsep* **(C)**, *Mrp2* **(D)**, *Ntcp* **(E)**, and *Oatp1b2* **(F)** in livers of the tested mice. The mRNA expression was analyzed by RT-qPCR and normalized to amplified β-actin. Data were expressed as mean ± SD (*n* = 7). *p < 0.05, **p < 0.01 *vs*. MCS group. ***p < 0.01 indicates statistically significant between MCS and MCD group.

The mRNA expression levels of hepatic BA metabolism- and transport-related genes of MCS, MCD, and SIL groups are shown in Figure 5. The mRNA expression levels of CYP7A1, CYP27A1, Bsep, Mrp2, Ntcp, and Oatp1b2 in the MCD group were significantly lower than those in the MCS group, but there was no significant difference compared with the SIL group.

### Effects of SIL on gut microbiota in mice

**Figure 6:**
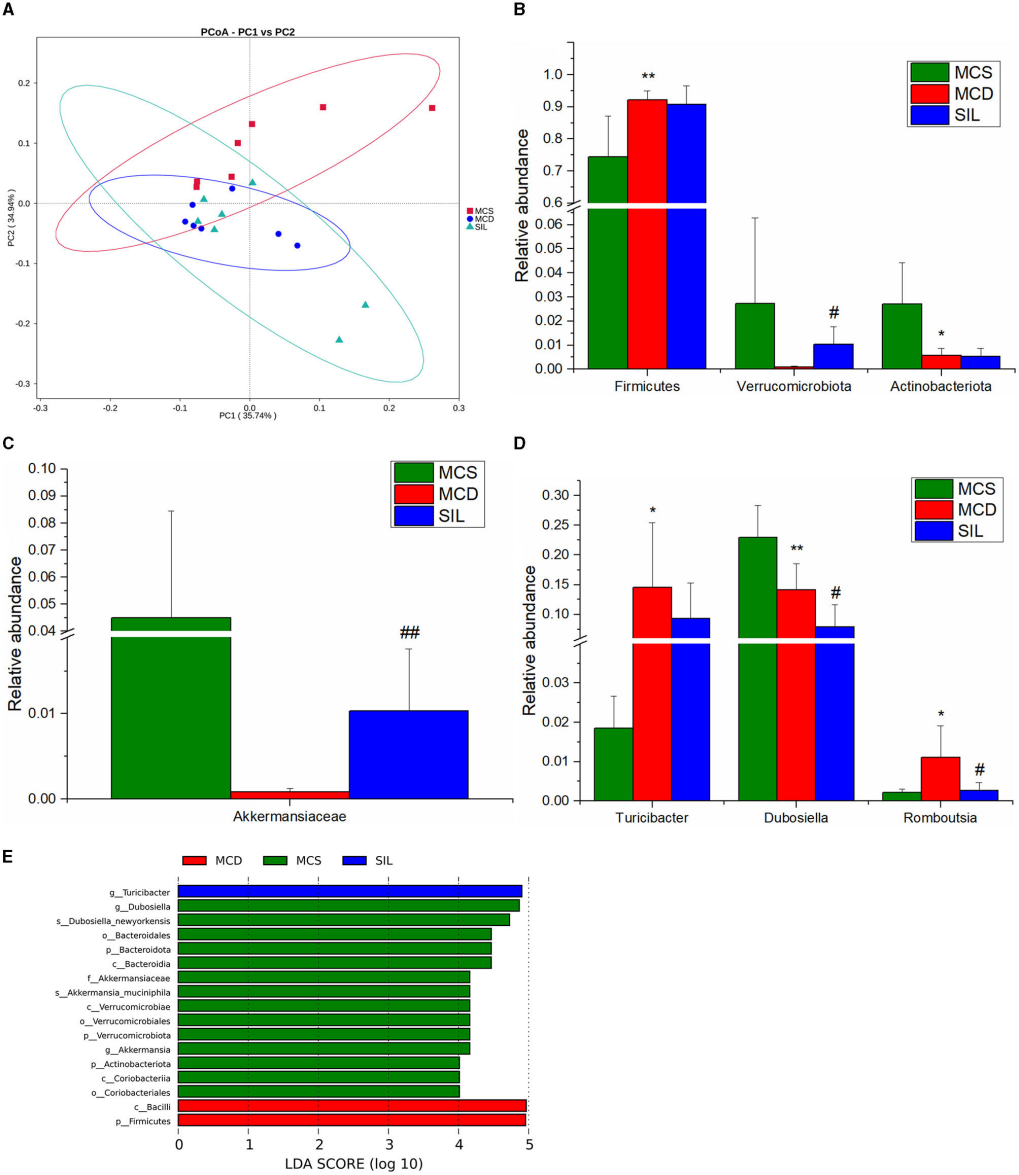
Effects of SIL on the composition of gut microbiota in tested mice (*n* = 7). **(A)** PCoA plot of gut microbiota based on weighted Unifrac metric. **(B)** Differential microbiota composition at the phylum level. **(C)** Differential microbiota composition at the family level. **(D)** Differential microbiota composition at the genus level. **(E)** LDA bar plot of LEFSe analysis on gut microbiota. *p < 0.05, **p < 0.01 indicate statistically significant between MCS and MCD group. #*p* < 0.05, ##*p* < 0.01 indicate statistically significant between MCD and SIL group.

The effects of SIL on microbes in the ileum of MCD mice were studied by 16S rDNA sequencing. The dilution curve analysis showed that the sequencing data in this experiment was progressive and reasonable and the sequencing depth was reliable. The statistical analysis results of alpha diversity index for the GM of different groups at the 97% consistency threshold showed a significant reduced Shannon index in the MCD group as compared with the MCS group, and no significant differences were observed for Chao1, ACE, Simpson, or Shannon indices between the SIL and MCD group, indicating that SIL treatment had no significant effect on intestinal microbial diversity. Therefore, the beta diversity analysis was performed to compare the composition of the GM of each group. Principal coordinate analysis (PCoA) results showed that the MCS group and the MCD group were significantly separated, and the SIL group partially overlapped with the MCD group, indicating that SIL caused a certain effect on the intestinal microbial disorder of MCD mice (Figure 6A).

The results of the phylum, family, and genera level analysis for GM composition of the three groups of mice are shown in Figures 6B–D. At the phylum level, the abundance of *Firmicutes* in MCD mice was significantly higher than that in the MCS group, while the abundance of *Actinobacteriota* was significantly lower than that in the MCS group. The SIL group showed significantly higher abundance of *Verrucomicrobiota* than that in the MCD group. At the family level, the abundance of *Akkermansiaceae* in the SIL group was significantly higher than that in the MCD group. At the genus level, the abundance of *Turicibacter* and *Romboutsia* in the MCD group was significantly higher than that in the MCS group, while the abundance of *Dubosiella* in MCD mice was significantly decreased compared to that in MCS mice. The SIL group showed lower abundance of *Dubosiella* than that in the MCD group. Linear discriminant analysis effect size (LEfSe) can identify the flora species with significant differences between groups (LDA value >4), and the histogram of LDA value distribution for LEfSe analysis of GM in the three groups is shown in Figure 6E.

### Spearman correlation analysis

**Figure 7:**
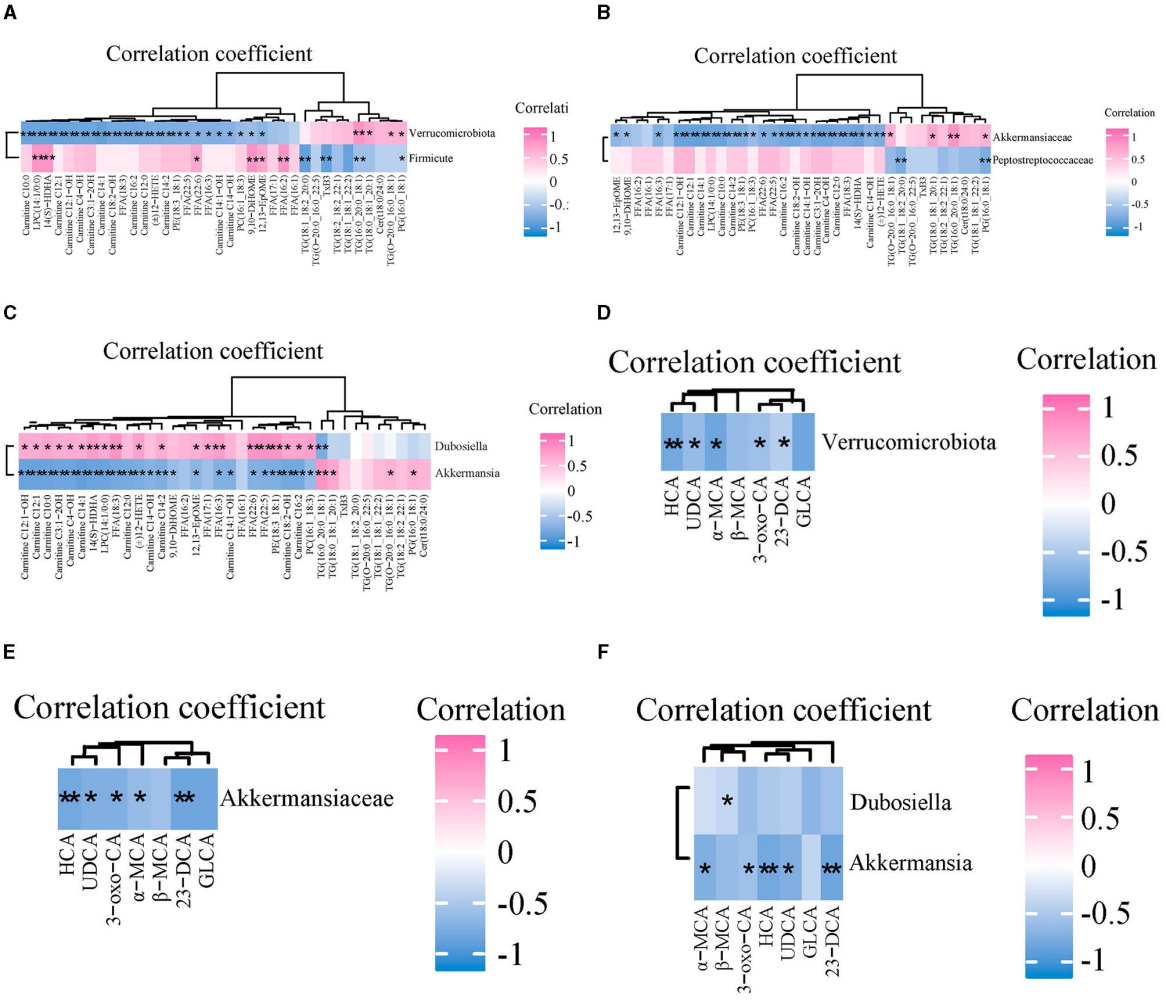
The correlations between gut microbiota and serum lipids and bile acids among three groups. **(A–C)** Spearman correlation analysis of gut microbiota and serum lipids at the phylum, family, and genus level, respectively. **(D–F)** The correlations between gut microbiota and serum BAs at the phylum, family, and genus level, respectively. *n* = 7. *p < 0.05, **p < 0.01 indicate that the correlation between the GM and lipids/BAs is statistically significant.

Spearman correlation analysis was used to explore the relationship between the abundance of GM and the content of serum lipid and bile acid in mice. The correlation between GM abundance and serum lipid levels is shown in Figures 7A–C. At the phylum level, *Verrucomicrobia* was negatively correlated with 12 ACs (C10:0, C12:1, C12:1-OH, C4-OH, C3:1-2OH, C14:1-OH, C16:2, C12:0, C14:2, C14:1-OH, C14-OH), four FFAs (18:3, 22:5, 22:6 and 16:3), three arachidonic acids [(±)12-HETE, 14 (S)-HDHA, 12,13-EpOME], and three GPs (LPC14:1/0:0, PE18:3_18:1, PC16:1_18:3), and positively correlated with three TGs (16:0_20:0_18:1, 18:0_18:1_20:1 and O-20:0_16:0_18:1) and one PG (16:0_18:1). At the family level, *Akkermansiaceae* was negatively correlated with four arachidonoids (12,13-EpOME, 9,10-DiHOME, 14 (S)-HDHA and (±)12-HETE), 12 ACs (C12:1-OH, C12:1, C14:1, C10:0, C14:2, C16:2, C18:2-OH, C14:1-OH, C3:1-2OH, C4-OH, C12:0, C14-OH), and four FFAs (16:3, 22:6, 22:5 and 18:3), 3 GPs (LPC14:1/0:0, PE18:3_18:1, PC16:1_18:3), and positively correlated with two TGs (18:0_18:1_20:1 and 16:0_20:0_18:1) and one PG (16:0_18:1). At the genus level, *Akkermansia* was consistent with *Akkermansiaceae* except that one TG (O-20:0_16:0_18:1) replaces another TG (18:0_18:1_20:1). In addition, *Dubosiella* was positively correlated with nine ACs (C12:1-OH, C12:1, C10:0, C3:1-2OH, C4-OH, C14:1, C14:2, C18:2-OH, C16:2), three Eicosanoids {14 (S)-HDHA, (±)12-HETE, 12,13-EpOME}, 5 FFAs (18:3, 17:1, 16:3, 22:6, 22:5), and three GPs (LPC14:1/0:0, PE18:3_18:1, PC16:1_18:3), and was negatively correlated with one TG (16:0_20:0_18:1).

The correlation between the abundance of GM and content of serum bile acids is shown in Figures 7D–F. At the phylum level, *Verrucomicrobia* was negatively correlated with UDCA, HCA, 3-oxo-CA, α-MCA, and 23-norDCA. At the family level, *Akkermansiaceae* was negatively correlated with HCA, UDCA, 3-oxo-CA, α-MCA, and 23-norDCA. At the genus level, *Akkermansia* was negatively correlated with 3-oxo-CA, α-MCA, HCA, UDCA, and 23-norDCA. *Dubosiella* was negatively correlated with β-MCA.

## Discussion

Excessive deposition of TG in the liver and high levels of FFAs in serum are the direct factors inducing NAFLD/NASH (17, 18). In addition, most fatty acid β-oxidative metabolism disorders and some organic acidemias can lead to abnormal accumulate of acylcarnitine, which has been associated with the development and progression of many diseases, such as inborn metabolic disorders, diabetes, and atherosclerosis (19, 20). In this study, SIL treatment significantly up-regulated the serum levels of seven unsaturated glycerides (TG16:0_20:0_18:1, 18:0_18:1_20:1, 18:1_18:2_20:0, 18:1_18:1_22:2, 18:2_18:2_22:1, O-20:0_16:0_18:1 and O-20:0_16:0_22:5), and down-regulated the levels of 12 serum acylcarnitines (AC-C4-OH, -C10:0, -C12:0, -C14-OH, -C3:1-2OH, -C12:1, -C12:1-OH, -C14:1, -C14:1-OH, -C14:2, -C16:2, -C18:2-OH) and TG in the liver of MCD mice, indicating that SIL inhibited TG production and promoted TG transport to the blood, while also reducing the content of harmful acylcarnitines in serum. Lysophosphatidylcholine (LPC) is a class of phospholipids that can not only induce endoplasmic reticulum stress by promoting the phosphorylation of eIF2α and expression of CAAT/enhancer binding homologous protein (CHOP), but can also induce caspase-dependent apoptosis in hepatocytes by activating the GSK-3/JNK pathway (21). The concentration of LPC in the liver tissue of NASH patients was higher than that of healthy controls (22). Oxidized lipids are a series of oxidative metabolites generated by automatic oxidation of polyunsaturated fatty acids (such as arachidonic acid, linoleic acid, α-linolenic acid, DHA, EPA, etc.) or under the action of specific enzymes (epoxidase COX, fat oxidase LOX, and CYP450); for example, 12,13-EpOME (23) and 9,10-DiHOME (24) are involved in the regulation of inflammatory response, immune defense, endocrine regulation and oxidative stress. Our results indicated that SIL treatment significantly reduced the serum levels of LPC (14:1/0:0) and oxidized lipids [12,13-EpOME, 9,10-DiHOME, (±)12-HETE, 14 (S)-HDHA], as well as the levels of liver MDA and serum ALT in MCD mice, indicating that SIL alleviated lipotoxicity and oxidative stress in the liver of MCD mice by decreasing the serum content of the above harmful lipids. Ceramide is a class of sphingomyelin with a 4,5-trans double bond produced by dihydroceramide desaturase (DES) 1 and 2. Saturated fat and HFD-induced hepatic steatosis and IR are closely related to an increased serum ceramide levels (25–27). Except for the upregulated serum Cer t18:0/24:0 level induced by SIL, no significant changes for the serum ceramide level among MCS, MCD, and SIL groups were found in this study, which could be caused by the differences in the composition of the MCD diet and HFD. In addition, KEGG pathway enrichment analysis showed that serum differential lipids in mice of the SIL and MCD groups involved 20 metabolic pathways, but only thermogenesis was closely related to the regulation of lipid metabolism disorders. Thermogenesis of brown and beige adipose tissue mediated by mitochondrial uncoupling protein 1 can convert chemical energy in fat cells into heat, which is important for maintaining normal cellular and physiological functions in warm-blooded animals under extreme environmental conditions. Therefore, the effect of SIL on brown and beige fat thermogenesis in MCD mice and its relationship with NASH deserve further study.

Patients with NAFLD have abnormal bile acid metabolism, such as significantly elevated bile acid levels in patients with NASH and mainly free CA and DCA (28). Serum levels of primary BAs increase as the severity of NASH increases (29). Studies showed that the hydrophobic LCA, DCA, CDCA, and GLCA were cytotoxic (30, 31), while the hydrophilic UDCA and TUDCA were cytoprotective (32). The present study found that SIL treatment up-regulated UDCA content and down-regulated 23-norDCA and GLCA levels in the serum of MCD mice, suggesting that SIL alleviated liver injury of MCD mice by reducing the production of harmful BAs. BAs are mainly synthesized in the liver by the rate-limiting enzymes CYP7A1 (classical pathway) and CYP27A1 (alternative pathway), while most of BAs are transported to the bile ducts by the bile salt export pump (BSEP) on the surface of hepatocytes. A small number of BAs are secreted into the bile duct by multi-drug resistance associated protein 2 (Mrp2) distributed in hepatocytes and stored in the gallbladder. BAs that flow into the liver through blood circulation are actively absorbed by BA transporters, including the Na^+^ taurocholate cotransporting polypeptide (NTCP) and organic anion transporting polypeptide (OATP), in the basal membrane of liver cells (29, 31). Our results found that the mRNA expression levels of CYP7A1, CYP27A1, Bsep, Mrp2, Ntcp, and Oatp1b2 in the livers of MCD mice were significantly lower than those of MCS mice, which was consistent with the literature (33). However, no significant difference on the mRNA expression of the above genes was observed between SIL and MCD groups, suggesting that SIL regulated the BA metabolism of MCD mice mainly by influencing GM and thereby by enterohepatic axis.

GM is involved in the metabolic regulation of many lipids, especially TG and LPC (34–36). *Akkermansia* is a genus of gram-negative anaerobic bacteria in the phylum *Verrucomicrobia*, and their abundance in the intestinal tract is significantly negatively correlated with obesity (37). Increasing the abundance of *Verrucomicrobia*, especially *Akkermansia* abundance, helped improve intestinal barrier function in obese mice (38) and inhibited the release of intestinal bacterial endotoxin LPS induced by a high-glucose-high-fat diet (39). HFD significantly inhibited the abundance of *Akkermansia* in the caecum of mice, and its abundance was positively correlated with fatty acid oxidation and fat browning and was negatively correlated with plasma markers of inflammation, lipid synthesis, IR, and obesity (40). The present study found that SIL treatment significantly increased the abundance of intestinal *Akkermansiaceae* in MCD mice, and its abundance was negatively correlated with four oxidized lipids [12,13-EpOME, 9,10-DiHOME, 14 (S)-HDHA, (±)12-HETE], 12 ACs (C12:1-OH, C12:1, C14:1, C10:0, C14:2, C16:2, C18:2-OH, C14:1-OH, C3:1-2OH, C4-OH, C12:0, C14-OH), and LPC14:1/0:0, and was positively correlated with two TGs (18:0_18:1_20:1 and 16:0_20:0_18:1) and one PG (16:0_18:1) (Figure 5C). These results suggested that SIL may reduce the serum content of toxic lipids by increasing the abundance of intestinal *Akkermansiaceae*, thereby alleviating liver steatosis and inflammation in MCD mice.

Metabolism of BAs by GM can regulate the levels of FXR agonists and antagonists, and can also produce non-bile acid-derived FXR ligands (41). Our results showed that SIL significantly increased the abundance of intestinal *Akkermansiaceae* in MCD mice, and its abundance was negatively correlated with serum levels of UDCA, 3-oxo-CA, and 23-norDCA, suggesting that SIL treatment affected the competition of bile acid agonists and antagonists for FXR signaling in ileal epithelial cells of MCD mice. In addition, SIL significantly inhibited the abundance of *Dubosiella* in the ileum of MCD mice, and the correlation between *Dubosiella* abundance and serum lipids or BAs was basically opposite to that of *Akkermansiaceae*. *Dubosiella* is a member of the *Erysipelotrichaceae* family and is a key bacterium for normal lipid metabolism. It has been reported that the abundance of intestinal *Dubosiella* is positively correlated with the improvement of glycolipid metabolism disorder in diabetic mice (42, 43) and weight gain in HFD mice and negatively correlated with the content of short-chain fatty acids in feces (44). Therefore, the relationship between intestinal *Dubosiella* abundance and NASH needs to be studied further.

## Conclusion

In this study, serum metabonomics and ileal 16SrDNA sequencing were conducted to investigate the underlying mechanisms of SIL in the prevention of NASH. We found that SIL had a good coordination in regulating the abundance of GM and the contents of serum lipids and BAs in MCD mice, that is, when the abundance of probiotics was up-regulated, the content of beneficial unsaturated fatty acids in serum was up-regulated, while the serum levels of harmful lipids and BAs were down-regulated. Therefore, the up-regulation of the abundance of ilium probiotic *Akkermansiaceae* and then down-regulation of the contents of harmful lipids and bile acids in serum were closely related to SIL-induced alleviation of NASH in MCD mice. Our results also indicated that SIL might serve as a promising dietary supplement for NASH prevention.

## Data availability statement

The datasets presented in this study can be found in online repositories. The names of the repository/repositories and accession number(s) can be found in the article/supplementary material.

## Ethics statement

The animal study was approved by Experimental Animal Ethics Committee of Hubei University. The study was conducted in accordance with the local legislation and institutional requirements.

## Author contributions

WW: Project administration, Writing—original draft. TZ: Project administration, Writing—original draft, Formal analysis. PL: Writing—original draft. XM: Writing—review and editing. JW: Writing—review and editing. YC: Writing—review and editing.
